# Panton-Valentine Leukocidin–Secreting *Staphylococcus aureus* Pneumonia Complicating COVID-19

**DOI:** 10.3201/eid2608.201413

**Published:** 2020-08

**Authors:** Claire Duployez, Rémi Le Guern, Claire Tinez, Anne-Laure Lejeune, Laurent Robriquet, Sophie Six, Caroline Loïez, Frédéric Wallet

**Affiliations:** Centre Hospitalier Universitaire Lille, Lille, France (C. Duployez, R. Le Guern, C. Tinez, A.-L. Lejeune, L. Robriquet, S. Six, C. Loïez, F. Wallet);; University of Lille (C. Duployez, R. Le Guern, A.-L. Lejeune)

**Keywords:** coronavirus disease, COVID-19, necrotizing pneumonia, Panton-Valentine leukocidin, severe acute respiratory syndrome coronavirus 2, SARS-CoV-2, Staphylococcus aureus, bacteria

## Abstract

Necrotizing pneumonia induced by Panton-Valentine leukocidin–secreting *Staphylococcus aureus* is a rare but life-threatening infection that has been described in patients after they had influenza. We report a fatal case of this superinfection in a young adult who had coronavirus disease.

Panton-Valentine leukocidin (PVL) is a cytotoxin produced by some strains of *Staphylococcus aureus*. These strains are responsible for primary skin infections and necrotizing pneumonia. This rare entity is mainly described in young immunocompetent patients with an influenza-like prodrome and has a high case-fatality rate (*1**,**2*). We report a case of necrotizing pneumonia induced by PVL-secreting methicillin-susceptible *S. aureus* in a patient infected with severe acute respiratory syndrome coronavirus 2 (SARS-CoV-2) and who had coronavirus disease (COVID-19).

In March 2020, during the SARS-CoV-2 outbreak in France, a man in his 30s who had no underlying conditions came to an emergency department because of fever, cough, and blood-streaked sputum that developed for 3 days. A diagnosis of pleuropneumonia was made, and antimicrobial therapy was initiated with cefotaxime plus metronidazole. Test results for *Streptococcus pneumoniae* and *Legionella pneumophila* serotype 1 urinary antigens were negative. A reverse transcription PCR specific for respiratory viruses also showed negative results.

The next day, further respiratory deterioration required transfer of the patient to an intensive care unit (ICU) for intubation, mechanical ventilation, and inotropic support. Spiramycin was added to the previous drug regimen. Chest computed tomography showed a parenchymal consolidation of the left upper lung without ground-glass opacities commonly described for COVID-19 (*3*).

Four days after intubation, the condition of the patient had not improved. We performed a reverse transcription PCR specific for SARS-CoV-2 on an endotracheal aspirate by using the method developed by the National Reference Centre for Respiratory Viruses (Institut Pasteur, Paris, France). The PCR result was positive for SARS-CoV-2 (*4*). Chest computed tomography showed worsening of bilateral parenchymal damage with complete consolidation of the left lung, cavitary lesions suggestive of multiple abscesses, and appearance of areas of ground-glass opacities in the right lung (Figure). The chest radiograph also showed a left pleural effusion.

**Figure:**
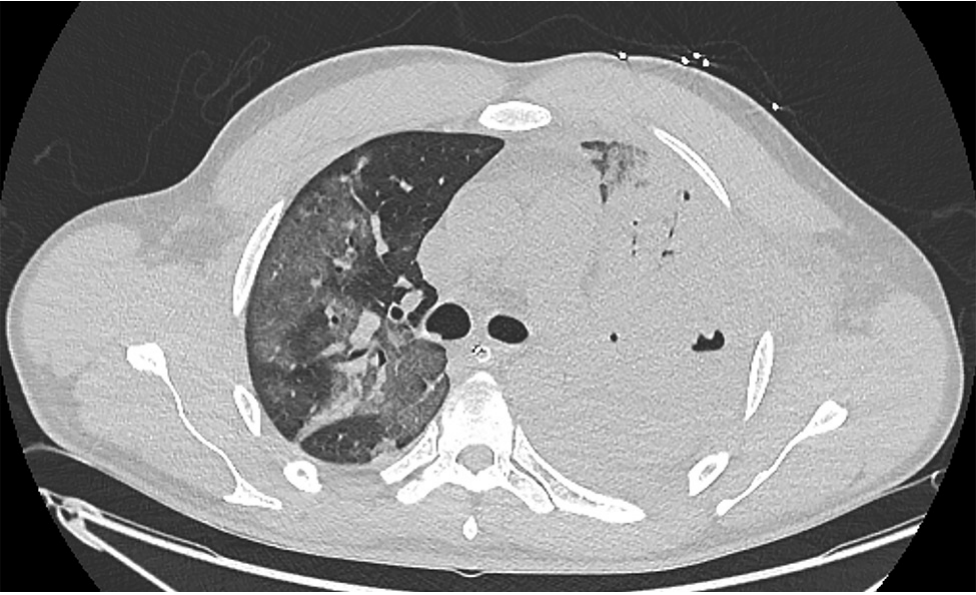
Chest computed tomography of a patient in France with Panton-Valentine leukocidin–secreting *Staphylococcus aureus* pneumonia complicating coronavirus disease, showing worsening of bilateral parenchymal damage with complete consolidation of the left lung, cavitary lesions suggestive of multiple abscesses, and appearance of areas of ground-glass opacities in the right lung

Bacteriological analysis of pleural drainage showed gram-positive cocci; the culture yielded monomicrobial *S. aureus*, which was identified by using matrix-assisted laser desorption/ionization time-of-flight mass spectrometry (Bruker Daltonics, https://www.bruker.com). The bacterial strain was resistant only to penicillin G (VITEK 2 System; bioMérieux, https://www.biomerieux.com). Because of this necrotizing pneumonia associated with acute respiratory distress syndrome, a PVL-producing strain was suspected. We confirmed PVL production by using a specific PCR as described by Deurenberg et al. (*5*).

We changed antimicrobial drug therapy to oxacillin plus clindamycin (for antitoxin effect) against methicillin-susceptible *S. aureus* and lopinavir/ritonavir (quickly stopped because of suspected toxicity) plus azithromycin against SARS-CoV-2. Three days later, given a lack of clinical improvement, antimicrobial therapy was changed to piperacillin/tazobactam plus linezolid (for antitoxin effect). Bronchoscopy showed that the left bronchial tree was obstructed by purulent secretions. Because of deterioration of respiratory, renal, and liver functions, venovenous extracorporeal membrane oxygenation and anticoagulation were initiated 10 days after ICU admission. Two days later, we performed upper left lobectomy, and antimicrobial drug therapy was incremented with meropenem, gentamicin, and linezolid. However, the patient died 17 days after his admission to the hospital.

PVL-secreting *S. aureus* necrotizing pneumonia is frequently preceded by an influenza-like infection (*6*), which might be a possible causative factor. Influenza virus is known to impede phagocytic killing and damage the bronchial epithelium, thus reducing secretin clearance and facilitating bacteria adhesion (*2*). It also induces an influx of immune cells to lung tissues, including neutrophils; the rapid killing of these cells by PVL and release of inflammatory mediators might promote disease development by damaging the epithelium (*7**,**8*). The association of PVL-secreting *S. aureus* and influenza virus has been reported (*6**,**9*). We report a PVL-secreting *S. aureus* superinfection in a patient who had COVID-19. Our findings indicate that the new SARS-CoV-2 is, in the same way, a facilitating factor for PVL-producing *S. aureus* necrotizing pneumonia.

In 2003, during the SARS-CoV outbreak, an increase in *S. aureus* superinfection (mostly methicillin-resistant *S. aureus* ventilator-acquired pneumonia) was described. Given common points between SARS-CoV-2 and previous coronaviruses, Lupia et al. discussed this issue for COVID-19 and suggested consideration of methicillin-resistant *S. aureus* coverage to reduce the risk of superinfection (*10*).

In PVL-producing *S. aureus* superinfection, prescribing antimicrobial drugs that have an antitoxin effect, such as clindamycin or linezolid, remains essential (*2*). Thus, in previously healthy young adults admitted to an ICU for COVID-19 and *S. aureus* superinfection, a PVL-producing strain should be assumed and treatment provided accordingly.
